# Assessment of Organic Matter Influence on the Ecological Integrity of Poyang Lake Using O/E Model and Chemical–Biological Indices over the Past Two Decades

**DOI:** 10.3390/toxics13010001

**Published:** 2024-12-24

**Authors:** Jindong Wang, Wenjie Huang, Chenglian Feng, Hongyang Wang

**Affiliations:** State Key Laboratory of Environmental Criteria and Risk Assessment, Chinese Research Academy of Environmental Sciences, Beijing 100012, China; wangjindong1103@163.com (J.W.); huangwenjie1210@163.com (W.H.)

**Keywords:** ecosystems, long time scale, benthic community, chemical–biological index

## Abstract

Ecological integrity, encompassing physical, chemical, and biological aspects, is crucial for sustaining ecosystem service functions and stability. As the largest freshwater lake in China, the ecological integrity of Poyang Lake has attracted much attention due to the over-exploitation of its water resources in recent years. In this study, several biological and water quality surveys on water ecological conditions were carried out at 11 sampling points of Poyang Lake from 1998 to 2022, and the ecological health of Poyang Lake was evaluated by use of the O/E (Observation/Expectation) model and the chemical–biological index method according to the status of the water quality and the structure of the benthic fauna in the four seasons, respectively. The results showed that the benthic community structure is simple, and the biodiversity is low, which is mainly dominated by Insecta in Arthropoda and Oligochaeta in Oroidea, accounting for 84.3% and 42.4%, respectively; the results of the O/E index evaluated under the threshold of probability of capture ≥ 0.5 showed that the health grade of all sections was sub-healthy or average, which was consistent with the results of the chemical–biological composite index evaluation. The dual evaluation method of the O/E model and chemical–biological composite index adopted in this study integrates more than twenty years of long-time scale data; this method combined with long time scale data has not been studied before, and its advantage is that it can more objectively show the change in the ecological situation of Poyang Lake for many years. The results of the present study could provide a theoretical basis and technical support for the evaluation of lake water environment quality.

## 1. Introduction

The Yangtze River, with a course of more than 6300 km, is the longest river in China and the core plate of central China [1]. Poyang Lake serves as a vital water source for local agriculture and industry. However, urbanization and road construction have significantly reduced the lake area, degraded lakeshore zones, and diminished suitable habitats for aquatic organisms [2]. Blockages in rivers and lakes due to lock and dam construction have reduced habitat heterogeneity. Excessive household sewage discharge has caused eutrophication and accumulation of harmful pollutants, including heavy metals. Overfishing of mussels and snails has depleted biological resources. As a key component of the river ecosystem, the structure and composition of aquatic communities integrate responses to physical, chemical, and biological influences across spatial and temporal scales, reflecting cumulative ecological stresses on the lake ecosystem [3,4]. At present, there are many methods to evaluate the ecological health of water bodies, but the principles of the evaluation methods can be broadly summarized into two categories: prediction models and multi-indicator system methods [5]. The River Invertebrate Prediction and Classification System (RIVPACS) is one of the most typical prediction modeling methods, and was firstly developed and established by the Institute of Freshwater Biology of the United Kingdom in 1984 [6]. This method mainly reveals the integrity of the biological composition of the river by constructing a model between the observed species richness (O) of the monitoring sites and the expected species richness (E) of the reference sites [7,8,9,10,11], and then reflects the health status of the river. However, this method only uses benthic animals as indicator organisms with certain limitations. Therefore, in this study, chemical indicators were added into the evaluation process, i.e., a combination of the O/E model and chemical–biological composite index was used to evaluate the ecological health of water in Poyang Lake. This method can not only improve the accuracy of the evaluation results, but also can diagnose the lake health problems more comprehensively.

Macrobenthic invertebrates are one of the most important representative groups of sedentary animals in aquatic environments, and they are in a key position in the aquatic food chain. Their life activities, such as feeding, burrowing, and pipe building, affect the decomposition of materials and energy flow in aquatic ecosystems, and the composition of their communities is extremely sensitive to changes in aquatic environmental conditions [12,13]. Therefore, macroinvertebrates are often used by researchers as indicator species of ecological health in the evaluation of lake health [14]. Many scholars have applied the macroinvertebrate integrity index (B-IBI) to evaluate the ecological health of watersheds; for example, Li [15] used the B-IBI method to evaluate the ecological health of the Qin River, a major tributary of the Yellow River; Huang [16] applied the B-IBI method to evaluate the environmental quality of the water body of the Ganjiang River system; at the same time, this method was applied in the Three Georges Reservoir, Weihe River Basin, Zhanghe River Basin, etc. [17,18,19]. But, these evaluations rely on abundant macrobenthic data, and predictive model1ing (O/E) has good results in case of insufficient data. Currently, Wu [20] has applied the O/E model to evaluate the ecological health of barrier shallow lakes in the middle and lower reaches of the Yangtze River, and Liu [21] has used the O/E model to evaluate the benthic integrity of the Huaihe River Basin. In the Baiyangdian Basin, Liu [22] integrated chemical indicators into the evaluation of the benthic ecological health of a variety of bioindicator species. And You [23] used benthic macroinvertebrates to carry out a biological integrity evaluation of the Poyang Lake wetland. Moreover, Chen [24] developed a novel indicator (Rtaw) to evaluate the ecological health of Poyang Lake.

In this study, we integrated the field survey data of Poyang Lake for more than 20 years (1998–2022), carried out a study of the O/E index based on the prediction model of the benthic fauna community, explored the method of evaluating the health of the lake with the O/E index and chemical–biological composite index, a combined evaluation method which had never previously been used in a study of Poyang Lake. Based on the abovementioned, the aims of the present study were as follows: (1) Evaluate the water ecological quality of Poyang Lake by using the O/E model; (2) Evaluate the water ecological health of Poyang Lake by calculating the chemical–biological composite index; (3) Construct the evaluation method of the O/E model and chemical–biological composite index by combining the two above mentioned methods, which will play a more comprehensive and objective role in the evaluation. This study can deepen the understanding of the degree of damage to the aquatic ecosystems as well as the change in the structure and function of benthic fauna at key sections of Poyang Lake, and the results can provide an important methodological reference for the evaluation of lake water environment quality.

## 2. Materials and Methods

### 2.1. Study Region

**Figure 1:**
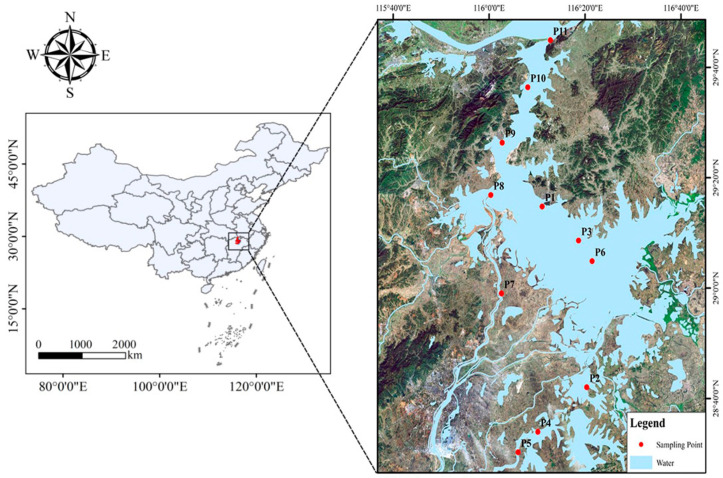
Poyang Lake (114°29′–118°12′ E and 26°50′–30°06′ N) is located in the north of Jiangxi Province, near the south bank of the middle reaches of the Yangtze River.

Poyang Lake (114°29′–118°12′ E and 26°50′–30°06′ N) is located near the south bank of the middle reaches of the Yangtze River, China (Figure 1) (Esri, ArcGIS 10.8, Redlands, CA, USA). It is the largest freshwater lake in China, and one of the only two large natural lakes remaining in the middle reaches of the Yangtze River. Poyang Lake belongs to the subtropical eastern monsoon climate zone, with obvious seasonal changes in temperature, average annual temperature of 17.6 °C, average annual precipitation of 1680 mm, and potential annual evapotranspiration of 1049 mm [25]. Poyang Lake has a zigzag shoreline with a maximum length of 170 km and an average width of 17 km, with a total basin of 16.2 × 10^5^ km^2^, accounting for 9% of the Yangtze River basin and 93.9% of the land area of Jiangxi Province [26]. Poyang Lake is the largest freshwater lake in China, and plays an important role in water resources, water environment, water ecology, and water safety in the middle and lower reaches of the Yangtze River, and is an important flood storage area and water resource recharge area [27]; it has an important international role in the wintering of migratory birds around the globe and the protection of dolphins [28,29].

### 2.2. Data Acquisition

#### 2.2.1. History Data

The benthic fauna and water quality data were based on our long-term research on Poyang Lake for more than 20 years and some data from the Institute of Aquatic Sciences, Chinese Academy of Sciences. Meanwhile, the natural predictors include geomorphological factors and climatic factors. The geomorphological factors of the lake include lake area, shoreline length, average water depth, average retention time of the lake, etc. The climatic factors are mainly derived from the average temperature, average rainfall, and bioclimatic data provided by WorldClim (https://worldclim.org/, accessed on 20 October 2024) from 1998 to 2022.

#### 2.2.2. Field Sample Collection and Processing

Water samples were collected at 0.5 m below the water surface in 500 mL polyvinyl chloride (PVC) bottles, a portable water quality meter (YSI, ProPlus, Yellow Springs, OH, USA) was used in the field to measure dissolved oxygen (DO), and a convenient ultrasonic detector (Speedtech, SM-5, Great Falls, VA, USA) was used to measure the water depth. Measurement of water samples collected was performed by adding the appropriate amount of toluene, adjusting the pH value of 1~2, protecting from light in refrigerated storage, and then transporting to the laboratory for water quality analysis. Total nitrogen (TN) was determined by alkaline potassium persulfate digestion and ultraviolet spectrophotometry (Thermo, AQ8100, Waltham, MA, USA), ammonia nitrogen (NH_3_-N) was determined by Nano reagent spectrophotometry, total phosphorus (TP) was determined by potassium sulphate digestion and ammonium molybdate spectrophotometry, and chemical oxygen demand (COD_Mn_) was determined by potassium permanganate method, and the specific methods for the analysis of the water quality were those referred to in the national standards (Methods for the Analysis and Detection of Water and Wastewater, Fourth Edition).

Benthic samples were collected along sampling transects using D-frame nets (30 cm diameter, 60-mesh aperture). Sampling occurred at wadable depths within 100 m river sections, with 3 to 10 quadrats sampled per point, proportionate to habitat conditions, resulting in a sampling area of approximately 0.9 to 3.0 m^2^. The benthic samples collected were first sieved through a 500 μm aperture sieve to wash off sludge and pick up inorganic impurities such as gravel, and then the samples were completely transferred to a sealed bag and added to a 75% ethanol solution. The biological samples were then completely transferred into sealed bags and fixed with 75% ethanol solution. If the presence of oligochaetes was initially identified by the naked eye, the biological samples were fixed with 7% formaldehyde solution and brought back to the laboratory for identification and analysis. The organisms were classified and counted under a laboratory stereomicroscope (LEICAMZ95). All organisms were identified to the lowest taxonomic unit, usually the genus level; however, mollusks were identified to species, oligochaetes to phylum, and aquatic insects to family or genus.

### 2.3. Construction of Assessment Method

#### 2.3.1. Construction of the O/E Index Model

Based on the RIVPACS model, the O/E index is obtained by measuring the species compositional integrity to carry out biological evaluation of water quality. The O/E index is the ratio of the true observed value of species richness (O) and the expected value of species richness (E) of the monitoring points to calculate the O/E index under different thresholds. The performance of the O/E index model is then compared under different thresholds, and the effect of rare species on the model is analyzed and excluded [30,31]. The O/E index model is constructed with reference to the existing more mature methods [32], and the specific calculation process is as follows: (i) the reference points are determined according to the physical and chemical conditions and habitat conditions of the points in Poyang Lake. The criteria for selecting the reference points are as follows: the area where the points are located is basically maintained in a natural state, the habitat conditions are good, and there is a certain area of submerged plants distribution; there is no or less pollution from surrounding surface sources; and there is no or less interference from aquaculture and fisheries. In addition, combined with the physical and chemical indicators, the points that did not meet the above conditions were classified as damaged points, and the severely damaged points were identified from the damaged points for the O/E index performance test. In addition, cluster analysis was used to distinguish the reference points into different point taxa based on the biological composition information of the reference points. And the benthic presence/absence data were used to calculate the S_Ørensen_ dissimilarity parameter for clustering, and then the flexibleβ (β = −0.5) method was used for clustering; all the points with a number of occurrences less than or equal to 5% were excluded from the clustering; (ii) Using the Random Forest model, natural predictors are used to predict the probability of monitoring points belonging to different reference point groups in (i); (iii) Using species presence/absence data, the probability of all species in the regional species pool belonging to each reference point taxon is calculated; (iv) the probability of capture (pc) for each species in the monitoring point is calculated by weighting the species probability in (iii) with the point probability in (ii)—the larger the pc value, the higher the probability of occurrence of the species, and the smaller the pc value, the lower the probability of occurrence of the species; (v) different pc thresholds are set (0 or 0.5) and the pc values of the species occurring in the monitoring point that are greater than the set pc thresholds are summed up to obtain the expected value of species richness (E) for the point, and at the same time, the pc values of all the real observed species in the point that are greater than the set pc thresholds are summed up to obtain the observed value of species richness (O) for the point; (vi) the O/E value is calculated, which is theoretically between 0 and 1; (vii) the degree of disturbance at the monitoring point is judged, i.e., when the O/E value deviates far from 1, it indicates that the point lacks some biological groups and is in a poor state of biological integrity. The Random Forest model was developed and implemented using PyCharm. During model optimization, 100 trees were selected, determined to be the optimal number based on preliminary testing. The maximum tree depth was set to the default value of None, allowing the trees to expand fully until the minimum sample split condition was satisfied. Model performance was optimized through the use of cross-validation to ensure robustness and generalizability.

The correspondence between the O/E scores and the health of the water body is shown in Table 1 [33].

#### 2.3.2. Calculation of the Biochemical Composite Index

The water ecological integrity is affected by many factors, such as land use status, water pollution status, hydrodynamic status, and human interference. Considering these influencing factors, combining domestic and international river health evaluation system construction methods, and according to the current ecological environment of Poyang Lake, the representative indicators were selected by the hierarchical analysis method to construct the evaluation index method for the water ecological health of Poyang Lake.

The system has two criteria layers: the chemical layer and the biological layer. The chemical layer mainly includes two factors, nutrient salts and oxygen balance, of which nitrogen and phosphorus are the main pollutants causing eutrophication in rivers. When the algae bloom, not only are the aquatic community structure and the number of species changed dramatically, destroying the balance of the aquatic ecosystem, but it is easy to change the original oxygen balance in the water body, disturbing the vitality of aquatic organisms and the self-purification ability of the water body. The high concentration of organic matter and ammonia nitrogen in the water body will easily consume the concentration of dissolved oxygen in the water, leading to the malodor of the river. Therefore, the inclusion of total nitrogen (TN), total phosphorus (TP), dissolved oxygen (DO), ammonia nitrogen (NH_3_-N), and permanganate index (COD_Mn_) in the ecological health evaluation system can help to improve the accuracy of the results. Macrobenthic invertebrates are key links in the aquatic food chain and are sensitive to changes in water conditions. Therefore, macrobenthic invertebrates were used as a factor in the biological layer, and the total number of taxonomic units (T), biodiversity index (H), and dominance index (D) were used as indicators in the health assessment system.

The above chemical and biological indicators are combined to calculate a composite index. First, to eliminate scale differences among indicators, each should be standardized. TN, TP, NH_3_-N, and COD_Mn_ indicators are standardized using Equation (1); DO indicators follow Equation (2); D indicators apply Equation (3); and T and H indicators are standardized using Equation (4). The calculation of the comprehensive index of health evaluation is obtained according to Equation (5), in which the factors of each indicator are calculated using equal weights, and the correction value is set by the weighted average method and the range of reference evaluation standards.

(1) $$S_{1}=\frac{C_{\max}-C}{C_{\max}-C_{\min}}$$

(2) $$S_{2}=\frac{C-C_{\min}}{C_{\max}-C_{\min}}$$

(3) $$S_{3}=\frac{Q_{95}-M}{Q_{95}-Q_{5}}$$

(4) $$S_{4}=\frac{M-Q_{5}}{Q_{95}-Q_{5}}$$

In the formula, *S*_1_, *S*_2_, *S*_3_, and *S*_4_ are the standardized values of each index; *C*_min_ and *C*_max_ are the minimum and maximum values of the corresponding indices of I~V in the environmental quality standard of surface water (GB3838-2002) [34]; *Q*_5_ and *Q*_95_ are the 5% and 95% percentiles of the biological data of all the sampling points; *C* and *M* are the measured values of water quality indices and biological indices of each monitoring point. *C* and *M* are the measured values of water quality indicators and biological indicators at each monitoring point.

(5) $$I=a\sum_{x=1}^{n}i_{x}$$

In the formula, *I* is the composite index value (0.8~1.0 for healthy, 0.6~0.8 for sub-healthy, 0.4~0.6 for general, 0.2~0.4 for poor, <0.2 for very poor); *i_x_* is the score value of the factor layer indicators; *a* is the corrected value, this paper calculates to take *a* = 0.5; for the two criterion layer, take *n* = 2; the specific calculations of the factor indicator layer are performed with Equations (6)–(9).

(6) $$i_{C}=i_{N}+i_{o}$$

(7) $$i_{N}=\frac{S_{TN}+S_{TP}}{2}$$

(8) $$i_{O}=\frac{S_{DO}+S_{NH_{3}-N}+S_{COD_{Mn}}}{3}$$

(9) $$i_{B}=\frac{S_{T}+S_{H}+S_{D}}{3}$$

where *i_C_* and *i_B_* are the scores for chemical and biological indicators, respectively; *i_N_* and *i_O_* are the scores for nutrient and oxygen balance indicators, respectively; and *S_TN_*, *S_TP_*, *S_DO_*, *S_NH3-N_*, *S_CODMn_*, *S_T_*, *S_H_*, and *S_D_* are the normalized values of the respective indicators.

## 3. Results and Discussion

### 3.1. The Composition and Structural Changes of Benthic Community in Poyang Lake over the Past 20 Years

**Figure 2:**
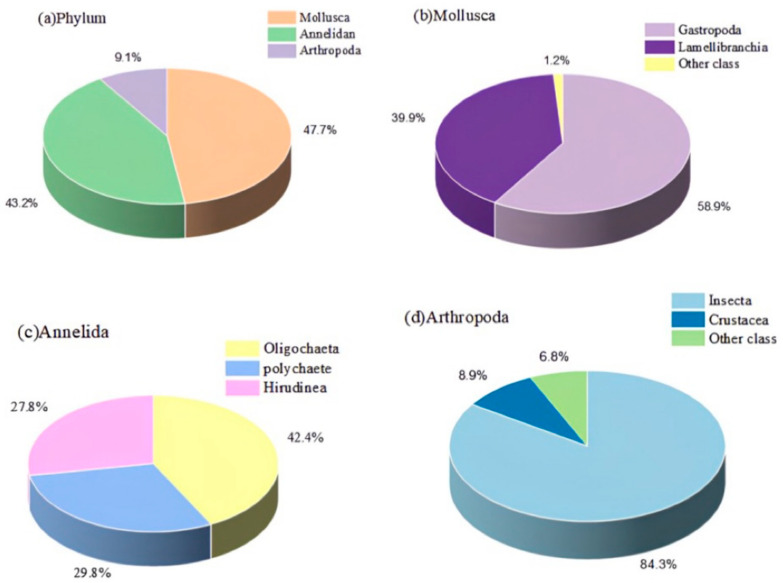
Benthic species representation diagrams. (**a**) Percentage of specific phyla in each of the three phyla, percentage of mollusks (**b**), annelida (**c**), and arthropoda (**d**).

The statistical results show that the proportions of Mollusca, Annelida, and Arthropoda in the total number of taxonomic units are 47.7%, 43.2%, and 9.1%, respectively. Specifically for the phylum Mollusca, Gastropoda and Valvularia occupied 58.9% and 39.9%, respectively, while the remaining phyla accounted for only 1.2%. In the phylum Annelida, oligochaetes, polychaetes, and vermiformes accounted for 42.4%, 29.8%, and 27.8%, respectively. In contrast, the phylum Arthropoda was dominated by Insecta with 84.3%, while Crustacea and other phyla accounted for 8.9% and 6.8%, respectively (Figure 2).

**Figure 3:**
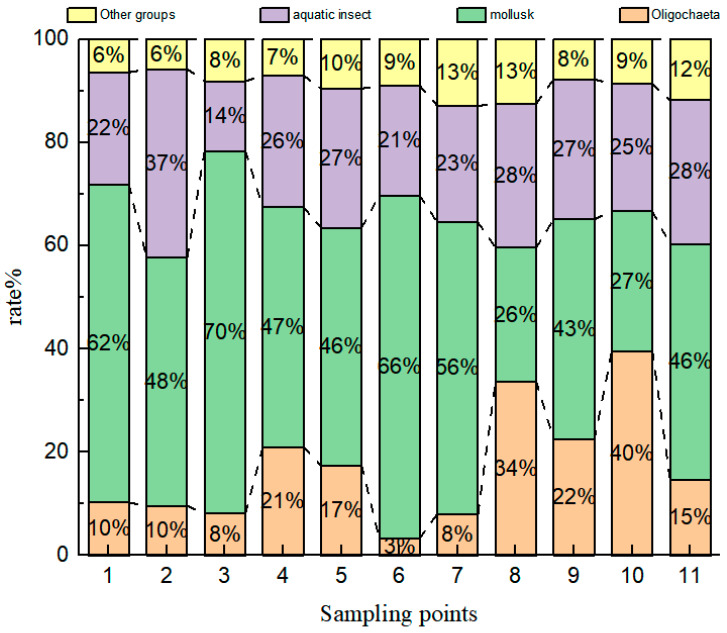
Histogram of “average” percentage distribution of macrobenthos at 11 sites “during the study period, from 1998 to 2022”.

In terms of the spatial distribution of sites (Figure 3), the distribution of mollusks was higher at all sites, especially at site P3, where it reached 70%; the distribution of oligochaetes as a whole was greater at the northwestern sites than the southeastern sites, i.e., the number at sites P8, P9, and P10 accounted for a greater proportion than the number at sites P2, P3, and P6; and aquatic insects and other species accounted for a relatively small proportion of the total number of sites.

The benthic community integrates physical, chemical, and biological influences across temporal and spatial scales, effectively reflecting cumulative ecological stresses on water health, and are among the most important indicator organisms for predicting changes in the quality of the aquatic environment and evaluating the health of rivers; the structural composition of the community and the abundance value of a single species vary significantly with changes in aquatic environmental factors. Domestic and international studies indicate that water quality deterioration significantly alters benthic community structure. Buss [35] identified fluctuations in dissolved oxygen as a primary driver of changes in benthic community structure; Bourassa and Morin [36] showed that benthic abundance varies with a change in the concentration of total phosphorus. Wu [37], in determining the riverine trophic salt concentration thresholds for macrobenthos in the upper waters of Xicamba Creek in the Taihu Lake Basin, found that total nitrogen and total phosphorus concentrations in the water column exceeded their respective thresholds, leading to severe degradation of benthic community structure; Wang [38] also pointed out that total nitrogen and total phosphorus were the main drivers of benthic community variability in the upstream watershed area of the Taihu Lake Basin. When the concentration of organic matter in the water body is too high, it will cause the concentration of dissolved oxygen in the water body to decrease, lead to the malodor of the river, and have a significant effect on the variation of the biological community structure. Therefore, for the water ecological problems faced by Poyang Lake, it is necessary to strictly control the input of point-source and surface-source pollution in the basin, and gradually improving the quality of the water body is the first priority; at the same time, habitat protection is also important for the protection of biodiversity.

### 3.2. Evaluation of Water Ecological Health Based on the O/E Index

**Figure 4:**
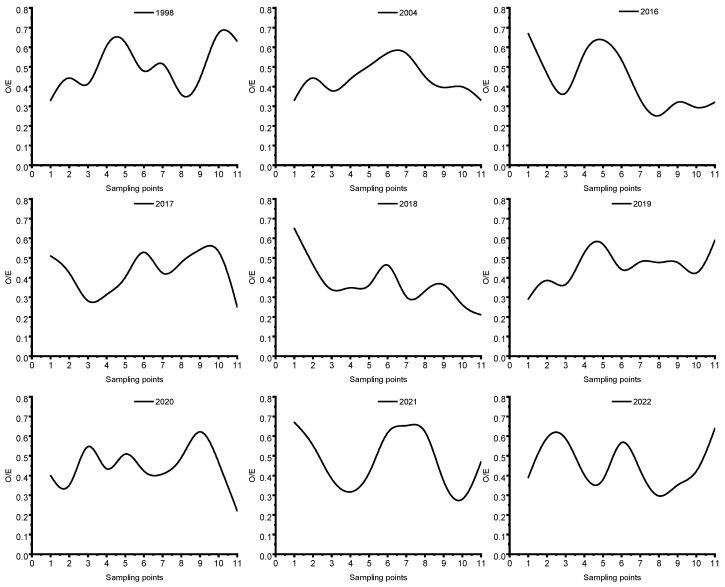
O/E index model score, as the ratio of observed to expected values, 1998–2022.

Deep analysis of the results of the O/E model’s evaluation of the ecological integrity status of 11 key cross-sections in Poyang Lake during the period from 1998 to 2022 reveals that the ecological health of these cross-sections has been largely maintained at a healthy or average level for more than two decades (Figure 4). This result reflects that the aquatic ecosystem in Poyang Lake has maintained a certain degree of self-repairing ability and stability in the face of multiple environmental pressures. However, when further observing the health status of these key cross-sections from the perspective of spatial distribution, it can be found that there are obvious differences. Among them, point P6, which is located in the area of Nanjishan Mountain in the center of Sanshan Lake, is particularly noteworthy, and its health status has been in a sub-healthy state for a long time. This result may be related to its unique geographical location and ecological environment. Since site P6 is located in the center of the lake, relatively isolated and less directly affected by human activities, its ecological health status may be more influenced by natural factors such as hydrological conditions and climate change. In addition, the area may lack the necessary anthropogenic management and ecological restoration measures, resulting in a relatively weak ecological self-repair capacity and difficulty in recovering to a healthier state. In contrast to point P6 is point P11. This point is located at the inlet of Poyang Lake, which is an important location where Poyang Lake meets the Yangtze River. Due to this special geographic location, the exchange of water bodies at point P11 is very frequent. When the water level of Poyang Lake is higher than that of the Yangtze River, the lake water will naturally flow into the Yangtze River through the river channel at the mouth of the lake; when the water level of the Yangtze River is higher than that of Poyang Lake, the water of the Yangtze River will be backed up and enter into Poyang Lake. This frequent exchange of water bodies makes the water quality and ecological health status of P11 site affected by multiple factors. On the one hand, the water exchange helps to dilute and diffuse pollutants, thus maintaining the relative cleanliness of the water body; on the other hand, when the Yangtze River water is backed up, it may bring pollutants and harmful organisms in the Yangtze River, which will have certain impacts on the water quality and ecosystem of Poyang Lake. Therefore, the health condition of point P11 is characterized by fluctuating changes.

The essence of the O/E index for the ecological health assessment of Poyang Lake is to compare the “distance” between the species richness composition of the monitoring point and the species richness composition of the reference point, which is less disturbed by human beings, but there are still many shortcomings in the practical application and its accuracy is affected by many objective factors [30,31]. In order to realize accurate evaluation of river health, in-depth research on technology and methodology is needed. Evaluation criteria, reference points, and biological data have always been the key factors for the accuracy of the ecological health evaluation results of the O/E model. In the evaluation process, the selection of reference points is a key factor for the accuracy of the results. Theoretically, uncontaminated areas with similar natural conditions are used as reference points, but it is difficult to find uncontaminated natural areas in China; furthermore, the number of reference points will also affect the accuracy of the evaluation results, and it has been shown that the greater the number of points, the more accurate the results will be [39]. At the same time, this method has been widely used in river management at home and abroad because it has the advantage of being applied to different ecological zones and can be used to evaluate the health of different river ecological zones as long as benthic organism data are obtained [40]. Liu and other scholars applied the O/E model and chemical–biological composite index to evaluate the ecological health of the Huaihe River Basin, and concluded that the deterioration of water quality is an important trigger for the sharp decline of species and is the main limiting factor for the poor ecological health of the water [21].

### 3.3. Combined Chemistry-Biology Index Health Assessment

**Figure 5:**
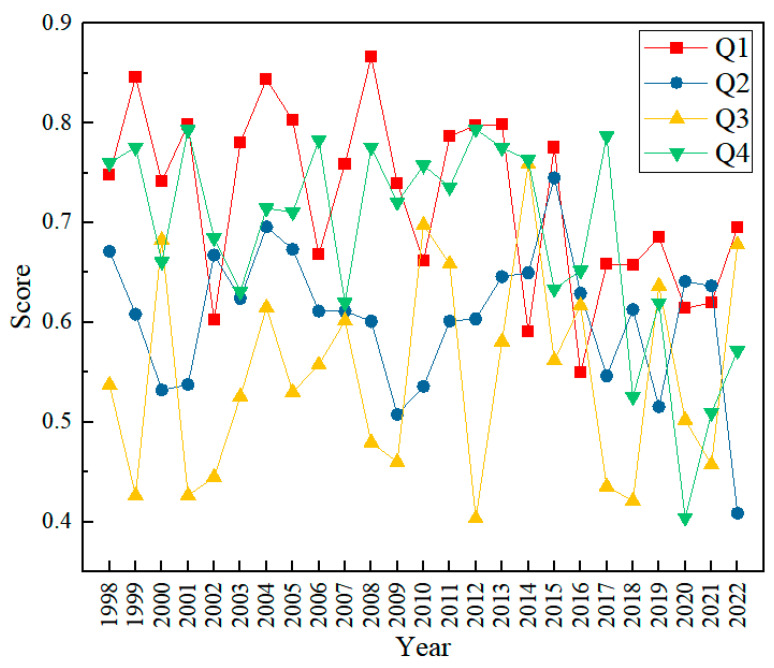
Combined Chemistry–Biology Index score, 1998–2022, January to March (Spring) as quarter 1 (Q1); April to June (Summer) as quarter 2 (Q2); July to September (Autumn) as quarter 3 (Q3); October to December (Winter) as quarter 4 (Q4).

Based on the results of the health evaluation of the chemical–biological composite index, the overall ecological health of the key cross-sections in Poyang Lake is in a sub-healthy and general state (Figure 5). This result reveals the serious challenges facing the water ecosystem in the basin. Going back to the time span of 1998–2022, although the health condition of the basin had once reached the healthy grade in certain time periods, it generally showed a deteriorating trend. This trend is undoubtedly closely related to the continuing impact of human activities, particularly industrialization, agricultural activities, and urbanization, where irrational development and pollution discharges have put significant pressure on water quality. With specific reference to seasonal variations, it can be noted that the health of the watershed is relatively good during the first quarter, especially in spring. This phenomenon is supported by obvious natural factors. Spring is the season of abundant rainfall, and the large amount of rainfall provides an effective replenishment of the water bodies, resulting in an enhanced self-purification capacity. At the same time, the dilution effect of rainwater on pollutants also reduces the concentration of pollutants, thus reducing the potential threat to the water ecosystem. As a result, water quality is relatively good in the spring and the health of the ecosystem improves accordingly. However, in the fourth quarter, especially in winter, the health status of Poyang Lake experiences significant fluctuations and declines. The low temperature and reduced precipitation in winter lead to lower mobility of the water body, and the diffusion and dilution of pollutants become difficult. In addition, winter is also a period of relatively concentrated human activities, such as agricultural irrigation and industrial discharges, which may further exacerbate the pollution of water quality. As a result, the state of water quality and ecosystem health is relatively poor in winter.

Meanwhile, it was found that the water quality of Poyang Lake was significantly affected by key factors such as total phosphorus (TP) during the calculation process. Especially during the flood season from May to October every year, the water quantity of Poyang Lake shows obvious seasonal fluctuations due to the increase in rainfall. This fluctuation not only affects the water level of the lake, but also produces a strong annual cyclical change on the water quality indicators. During the flood season, the level of organic pollution in the water body increased significantly with the increase in rainfall. This is due to the increase in surface runoff caused by the scouring action of heavy rainfall, and a large amount of suspended sediment is carried into the lake. These suspended sediments are rich in phosphorus and other nutrients, and when they are released into the water, they lead to an increase in the phosphorus concentration in the water body, thus exacerbating the degree of eutrophication in the water body. From the point of view of spatial distribution, the water quality condition of the waterways entering the river from the southeastern to the northern part of the lake improved during the flood season. This may be attributed to the higher mobility and dilution effect of the inlet waterways, which can effectively reduce the concentration of pollutants. Comparatively speaking, the nitrogen and phosphorus concentrations in the center of the lake were kept at a lower level with less fluctuation. This may be due to the fact that the water flow in the center of the lake is relatively slow, and pollutants are not easily spread, while the ecological environment in the center of the lake is relatively good and has a certain self-purification ability. However, it is worth noting that under the scenario of heavy rainfall during the flood season, the magnitude of change in the concentration of nitrogen, phosphorus, and other pollutants out of the lake was more significant compared to the non-flood season [39]. This may be due to the fact that the scouring effect of heavy rainfall resulted in a large release of nutrients such as nitrogen and phosphorus from the soil into the water body, while the increase in the water volume of the lake accelerated the diffusion and migration of pollutants, leading to the frequent occurrence of ecological problems such as cyanobacterial blooms, which had a long-term impact on the balance and stability of the lake ecosystem [21].

Meanwhile, the excessive use of agricultural nitrogen fertilizer is one of the important factors leading to the decline of water quality in Poyang Lake. In agricultural production, the excessive application of nitrogen fertilizers not only wastes resources, but also enters the rivers through the drainage system of farmland and eventually sinks into the lake. When the nitrogen in these nitrogen fertilizers enters the water body, it will promote the excessive reproduction of algae and other plankton, leading to eutrophication of the water body. In addition, the direct discharge of domestic sewage is also an important factor affecting the water quality of Poyang Lake. Urban and rural domestic sewage contains a large amount of organic matter and nutrients such as nitrogen and phosphorus, and if it is directly discharged into the river without treatment, it will have a serious impact on the water quality of the lake. In addition, the release of soil phosphorus caused by heavy rainfall washout is also an important factor affecting the water quality of Poyang Lake. Under heavy rainfall weather, rainwater will wash the ground and bring the phosphorus elements in the soil into the river. When these phosphorus elements enter the water body, they will likewise promote the propagation of algae and other plankton and aggravate the eutrophication of the water body.

In order to effectively deal with these surface-source pollution problems and protect the water quality and ecological environment of Poyang Lake, a series of effective measures need to be taken. First of all, it is key to strengthen the monitoring and management of the water quality of the rivers entering the lake. It is necessary to establish a perfect monitoring network to monitor the water quality of the rivers entering the lake in real time and take timely measures to control the pollution problems. At the same time, it is necessary to strengthen the ecological restoration of rivers, improve the self-purification ability of rivers, and reduce the number of pollutants into the lake. Secondly, controlling agricultural surface pollution is also very important. We need to promote scientific agricultural planting techniques, reduce the excessive use of nitrogen fertilizers, and improve the utilization efficiency of nitrogen fertilizers. At the same time, we should strengthen the construction and management of farmland drainage systems and prevent farmland drainage from being discharged directly into rivers. In addition, promoting environmentally friendly agricultural models such as ecological agriculture and organic agriculture is also an effective way to reduce agricultural surface source pollution. Finally, improving the efficiency of sewage treatment is also an important measure to protect the water quality of Poyang Lake. We need to strengthen the construction and renovation of urban and rural sewage treatment facilities to improve the capacity and efficiency of sewage treatment. At the same time, in addition, the operation and management of sewage treatment facilities must be strengthened to ensure the normal operation of the facilities and that the sewage treatment effect meets the standard. In conclusion, the protection of water quality and the ecological environment of Poyang Lake requires the joint efforts and continuous investment of the whole society. Effective measures should be taken to deal with the problem of surface pollution, strengthen water quality monitoring and management, control agricultural surface pollution, improve the efficiency of sewage treatment, etc., to ensure that the water quality and ecological environment of Poyang Lake are effectively protected and restored.

Many researchers have carried out studies on the changes of benthic community structure and diversity from the aspects of climatic conditions and land use [41,42], and initially established the quantitative relationship between them and the organisms, but the quantification is not deep enough, and it still needs to be combined with the chemical indices in order to elucidate the relationship. Tao [43] pointed out in their study on the ecological health of the Shaying River Basin using the chemical–biological index method that urban and industrial discharges are the main cause of the ecological health of the area. If the biological data represent all the ecosystem services of the river, it is one-sided and easy to ignore the ecological pressure that is not reflected in the benthic organisms. Therefore, in the process of river health evaluation, combining water quality and biological data, screening representative indicators to construct a comprehensive evaluation system, helps to improve the accuracy and comprehensiveness of the evaluation results. This method is more comprehensive and flexible than the O/E model, and avoids the errors caused by the constant benthic rating criteria in the O/E model. Therefore, the use of the O/E model and chemical–biological index evaluation method in Poyang Lake has good complementarity and can reflect the ecological health status more comprehensively.

## 4. Conclusions

In this study, the O/E model and the chemical–biological integrated index evaluation method were used to evaluate the water ecological quality of Poyang Lake with more than twenty years of data (1998–2022), and the case study showed that the ecological health evaluation results of the O/E model in the period of 1998–2022 showed that the health status of the eleven key sections in Poyang Lake was in the sub-healthy or general status in the whole year. At the same time, the health evaluation results of the chemical–biological integrated index in the period of 1998–2022 showed that the health status of the eleven key sections in Poyang Lake showed a trend of the first quarter > fourth quarter > second quarter > third quarter. The health evaluation results of chemical–biological composite index from 1998 to 2022 showed that the health status of Poyang Lake showed the trend of the first quarter > fourth quarter > second quarter > third quarter, and the evaluation status was sub-healthy or general except the first quarter of the five years of 1999, 2003, 2005, 2009, and 2012, which was excellent. Finally, the results of O/E model health evaluation and chemical–biological composite index health evaluation results are overall consistent, with good complementarity and mutual testing. Protecting biodiversity is likely a key factor in restoring Poyang Lake’s aquatic ecosystem health. This is because these biological components play a vital role in maintaining the ecological functioning of the lake, increasing the resilience of the ecosystem and restoring its natural ecological balance. At the same time, the results of the study can provide a theoretical basis and technical support for the evaluation of the quality of the lake water environment.

## Figures and Tables

**Table 1:** O/E index system evaluation criteria.

Level	O/E
Excellent	≥0.79
Healthy	0.59~0.79
Sub-healthy	0.39~0.59
General	0.19~0.39
Poor	≤0.19

## Data Availability

Data are contained within the article.
